# Immunomodulatory Significance of Mast Cell Exosomes (MC-EXOs) in Immune Response Coordination

**DOI:** 10.1007/s12016-025-09033-6

**Published:** 2025-02-20

**Authors:** Daniel Elieh-Ali-Komi, Farzaneh Shafaghat, Shamila D. Alipoor, Tohid Kazemi, Dmitrii Atiakshin, Polina Pyatilova, Marcus Maurer

**Affiliations:** 1 Institute of Allergology, Charité – Universitätsmedizin Berlin, Corporate Member of Freie Universität Berlin and Humboldt-Universität Zu Berlin, Berlin, Germany; 2 Fraunhofer Institute for Translational Medicine and Pharmacology ITMP, Immunology and Allergology, Berlin, Germany; 3 Immunology Research Center, Tabriz University of Medical Sciences, Tabriz, Iran; 4 Department of Immunology, Tabriz University of Medical Sciences, Tabriz, Iran; 5 Division of Inflammation and Infection, Department of Biomedical and Clinical Sciences, Linköping University, Linköping, Sweden; 6 Research and Educational Resource Center for Immunophenotyping, Digital Spatial Profiling and Ultra-Structural Analysis Innovative Technologies, Peoples’ Friendship University of Russia, 6 Miklukho-Maklaya St, 117198 Moscow, Russia; 7 Research Institute of Experimental Biology and Medicine, Burdenko Voronezh State Medical University, 394036 Voronezh, Russia

**Keywords:** Mast cell, Exosome, FcεRI, Extracellular vesicles, MiRNA, CD63

## Abstract

Mast cells (MCs) communicate with other cells by direct cell-to-cell interaction, secreting mediators, and releasing exosomes (EXOs). MC-exosomes (MC-EXOs) contain proteins, lipids, mRNAs, and noncoding RNAs (ncRNAs), exhibit typical EXO markers such as heat shock proteins, tetraspanins, tumor susceptibility gene 101 protein (TSG101), and ALG-2-interacting protein X (ALIX), and are released constitutively or following MC degranulation. MC-EXOs also have signature MC markers like FcεRI and KIT (CD117), which allows for their identification and comparison with other EXO populations. Following their release, MC-EXOs may interact with the recipient cell(s) directly or be internalized and then release their protein and nucleic acid content. This may contribute to the regulation of immune responses and other biological processes and reprogramming of recipient cells. MC-EXO proteins may integrate and become a functional part of the recipient cell membrane. The mRNA transferred by MC-EXOs is functional and the transfer of exosomal RNA to other MCs results in the expression of donor MC proteins in the recipient MCs. Moreover, MCs may function as the recipients of EXOs that are released by other non-immune and immune cells, altering the secretome of MCs. In this review, we focus on how MC-EXOs modulate the biology of other cells and vice versa; and we highlight the role of MC-EXOs in the pathogenesis of allergic and non-allergic diseases.

## Introduction

Mast cells (MCs) are CD117^+^/FcεRI^+^ tissue-resident immune cells [1–3] best known for their central role in allergic diseases [4–7]. They are also held to contribute to the pathophysiology of non-allergic conditions including cancers, infertility, and impaired wound healing [8–20]. MCs become activated via several pathways which include IgE: FcεRI, C5a: C5aR, and neuropeptide engagement of Mas-related G protein-coupled receptor X2 (MRGPRX2) [21–26]. This results in their degranulation and release of a plethora of preformed mediators including histamine as well as the de novo production and secretion of cytokines and growth factors [27–29].

It is important to note that the type and expression levels of these receptors differ not only between human and mouse MCs (MrgprB2 is the mouse counterpart (ortholog) of MRGPRX2 [30]) but may also vary depending on the specific organ from which the MCs are harvested within a given species (fresh human skin MCs have high MRGPRX2 expression levels which decrease over time in vitro [31]) Additionally, cell lines may differ in their expression profiles of activating receptors, resulting in different responses to various activators. For instance, between the two human mast cell lines, LAD-2 and HMC-1, only LAD-2 cells express FcεRI [32–35]).

MCs are preferentially located in the skin, gut, and airways, where their activation and degranulation results in local inflammatory reactions [36–38]. For their communication with other cells, MCs use direct cell-to-cell interaction, the secretion of mediators, and the release of EXOs [39]. EXOs are lipid bilayer cup-shaped vesicles, 30–100 nm in diameter, released by many cells in vivo and in vitro [40]. The latter mechanism likely provides a highly stable microenvironment to transfer the mediators through circulation due to its protective bilayer structure [41].

They originate from the internal vesicles of multivesicular bodies (MVBs), which are generated by the inward budding of their limiting membrane, resulting in a lumen content that is comparable to that of cytoplasm [42, 43].

**Fig. 1:**
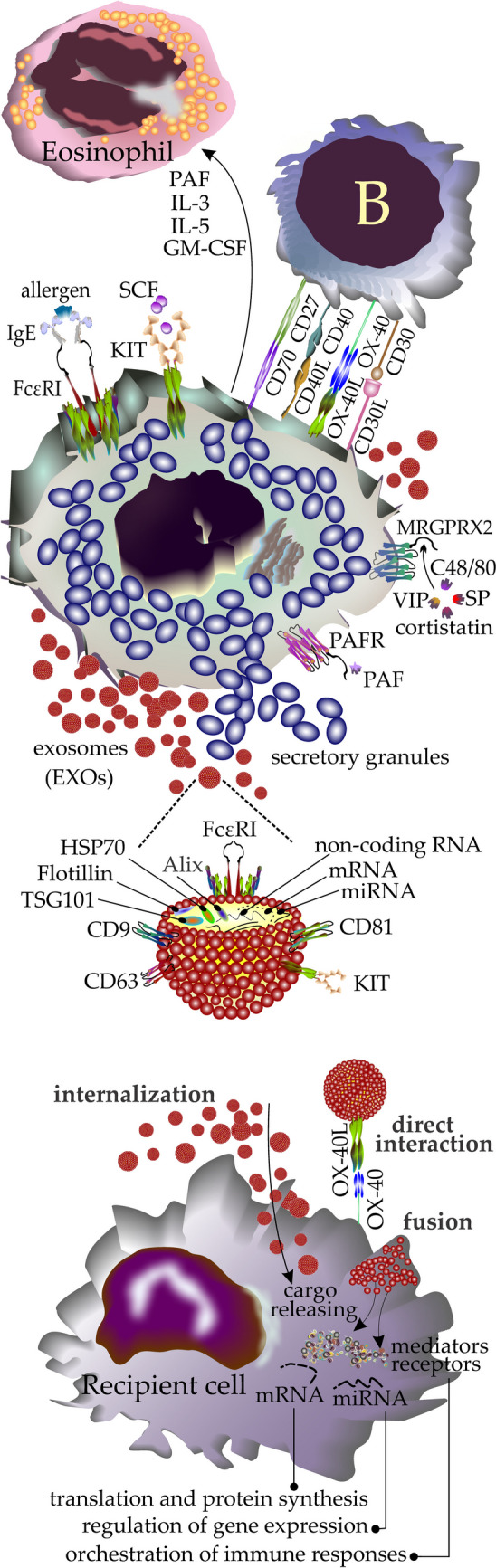
MCs employ a variety of mechanisms to communicate with other cells including the production and release of EXOs (MC-EXOs). MCs are activated when receptors like FcεRI, MRGPRX2, KIT, and PAFR are engaged by their corresponding ligands shown in the figure. MCs use direct cell-to-cell interaction or cytokine release to crosstalk with other cells (their interaction with B cells and eosinophils respectively is depicted). The third mechanism used by MCs to remotely interact with other cells is the production and release of MC-EXOs. They contain common EXO markers including HSP70 and TSG101 and RNAs including mRNAs, and noncoding RNAs (ncRNAs). Additionally, they may carry MC signatures like KIT or subunits of FcεRI. MC-EXOs after being released use different mechanisms to deliver their content or message including (a) direct interaction with recipient cell surface receptors that results in triggering downstream signaling, (b) internalization: EXOs are commonly internalized by the recipient cells through a variety of molecular mechanisms. (c) Fusion: EXOs may fuse with the plasma membrane and, like the internalization mechanism, release their cargo into the cytoplasm. The Molecules liberated from EXOs into cytosol act differently to exert their biofunction in the recipient cell and modulate physiologic processes such as producing new proteins, regulation of gene expression, and orchestration of immune responses. *MRGPRX2* mas-related G protein-coupled receptor X2, *PAFR* platelet-activating factor receptor, *SP* substance P, *VIP* vasoactive intestinal polypeptide, *ALIX* ALG-2-interacting protein X, *TSG101* tumor susceptibility gene 101 protein

EXOs contain nucleic acids, proteins, and lipids, which vary in quantity and type depending on the source cell [44]. EXOs eventually fuse either with lysosomes, leading to the degradation of EXO, or with the cell membrane. The latter is known as exocytosis and results in the release of EXOs from the cell [45]. Owing to their endosomal origin, EXOs contain common membrane transport and fusion proteins including GTPases, annexins, and flotillin. Molecular investigations showed that EXOs have a shared profile of tetraspanins (CD9, CD63, CD81, and CD82) and heat shock proteins (Hsp70 and Hsp90) [46]. Typical EXO markers include CD63, TSG101, HSP70, and Alix, whereas proteins including calnexin are used as negative markers to distinguish EXOs from other vesicles or contaminants [47–49] (Fig. 1).

There are traditional isolation methods for EXO isolations including differential ultracentrifugation (considered as “gold standard” method, Principle: size and density, Purity: medium), density gradient centrifugation (Principle: size and density, Purity: high), size-exclusion chromatography (Principle: size, Purity: high), ultrafiltration (Principle: size, Purity: low), immunoaffinity capture (Principle: specific binding, Purity: high), and precipitation (Principle: solubility, Purity: low). Currently, emerging other techniques and methods like microfluidics-based isolation techniques, immunomagnetic beads, covalent chemistry, and DNAzyme probes has effectively improved the efficiency of EXO detection and/or isolation [50–56].

In mast cell biology, EXO release and degranulation share certain similarities but also exhibit distinct differences. Degranulation is a rapid, large-scale process involving the release of pre-formed granules containing mediators such as histamine and proteases, primarily driving acute inflammatory responses[10]. In contrast, EXO release is a more controlled and sustained process, involving small vesicles that selectively carry proteins, lipids, and nucleic acids, contributing to long-term intercellular communication. While degranulation is typically triggered by the binding of an activator, such as IgE, to its corresponding receptor followed by downstream signaling events, EXO release is not entirely dependent on this pathway, although it can be accelerated by mast cell activation. Furthermore, degranulation involves the fusion of visible granules with the cell membrane, whereas EXO release occurs on a smaller, more specialized scale through the secretion of vesicles derived from multivesicular bodies [57, 58].

The production of MC-EXOs may require cytokines. For instance, murine bone marrow-derived MCs (BMMCs) require IL-4 to produce EXOs [59] while MC lines like RBL-2H3, P815, and MC/9 constitutively secrete EXOs [39, 41, 60]. Therefore, even resting MCs have a basal level of EXO release which is induced by treating MCs with secretagogue ionomycin in vitro or through the IgE-FcεRI pathway [61]. MC-EXOs are immunologically active and modulate the biology of T and B cells including blast formation, proliferation, and production of IL-2 and IFN-γ [59].

Here, we review (a) the cargo of MC-EXOs, (b) the immunological effects of MC-EXOs, and (c) the role and relevance of MC-EXOs in the pathogenesis of MC-driven diseases. We also highlight questions on MC-EXOs that remain to be answered. To achieve this, we discuss the results obtained from various human mast cell lines (HMC-1, LAD2, LUVA), as well as mouse (MC/9, P815) and rat (RBL-3) cell lines, alongside organ-derived mast cells from both human and mouse tissues [62, 63]. Finally, we have addressed the different types of extracellular vesicles (EVs), including EXOs, based on their usage in the original studies and their classification criteria (such as size, origin, and other factors) to ensure they are not used interchangeably.

## Comparative Analysis of Genetic and Immunologic Profiles of MC-EXOs

### Marker Characterization of MC-EXOs

Structurally, MC-EXOs are made up of a lipid bilayer equipped with surface receptors/ligands that hold various bioactive molecules including proteins, RNAs, and DNAs. MC-EXOs acquire all of their cargo from their parental cells, they may contain molecules later internalized in vitro acting as antigens such as transferrin, bovine serum albumin (BSA), and ovalbumin (OVA) once they are preincubated overnight with these antigens [60]. When MC-EXOs are exposed to the host’s immune cells in vivo and in vitro, the exosomal antigens are recognized [60]. Of note, MC populations differ in their MC-EXOs content Table 1.

**Table 1:** List of MC-EXO contents derived from human/rodent MC lines and tissue-derived MCs

Human cell lines				Human Tissue-derived MCs	Mice		
HMC-1	HMC-1.1	HMC-1.2	LAD2	Placental MCs	MC/9	RBL (rat)	mBMMC
CD63 [64]	CD63 [65]	CD63 [65]	KIT [65]	CD63 [66]	CD63 [67]	CD63 [61]	MHC II [68]
KIT [64]	CD81 [65]	CD81 [65]	CD63 [69]	HSP70 [66]		MHC II [61]	CD13 [68]
Factor V precursor [70]	CD9 [65]	CD9 [65]	PLA2 [69]	Calnexin (low) [66]		CD81 [61]	ribosomal protein S6 kinase [68]
Prothrombin [70]	flotillin‐1 [65]	flotillin‐1 [65]				phospholipases (A2, C, and D) [71]	annexin VI [68]
Angiotensinogen [70]	ALIX [65]	ALIX [65]				aldolase A	CDC25 [68]
TNF-α precursor [70]	syntenin‐1 [65]	syntenin‐1 [65]				Hsp 70	γ actin-like protein [68]
CD81 [64]	TSG101 [65]	TSG101 [65]				arachidonic acid	γ-actin [68]
TSG101[64]	KIT (higher than HMC-1.2) [65]	KIT [65]				PGE 2	CD63 [46]
	Tryptase (higher than HMC-1.2)	Tryptase [65]				15-d PGJ 2	OX40L [46]
						Hsc70	hsp60 [60]
						Hsp90-β	hsc70 [60]
						Hsp 70	FcεRI [72]
						Transferrin receptor	
						Casein kinase II subunit	

*mBMMC* mouse bone-marrow-derived mast cells, *15-d PGJ 2* 15-deoxy-Δ^12,14^-prostaglandinJ2.

MC-EXO profiling has several benefits: (1) it may be used to ascertain the potential consequence of EXO uptake and its effect on the target cell(s); (2) it can be used to identify the cells from which the EXOs have been generated. The latter becomes of particular interest to identify the biological source of a particular EXO in a complicated media, such as serum. In line with this, Kim et al. have shown that extracellular vesicles (EVs) in the serum of patients with systemic mastocytosis have MC signatures including KIT, FcεRI, MRGX2, and tryptase, suggesting that MC is the main source of EVs in these patients [73]; (3) it provides interesting information regarding the biogenesis and intracellular delivery of MC-EXOs molecules from other compartments and organelles. In one immunophenotypic-based study, the strength of the signal (positivity) determining CD63 location in granules of MCs collected from patients with melanoma was monitored. According to the results, MCs’ strong immunopositivity to CD63 was found around the granules’ periphery suggesting that mature granules could be a source of specific proteases when MC-EXOs are formed [74]; (4) mRNA profiling of MC- EXOs may determine the functional mRNA responsible for expression of new proteins which necessarily are not present in EXOs. In this regard, mouse MC/9 cell line-derived EXOs transfer functional mRNAs to code Cox5b, Hspa8, Shmt1, Ldh1, Zfp125, Gpi1 and Rad23b proteins [67].

### microRNA Content of MC-EXOs

**Fig. 2:**
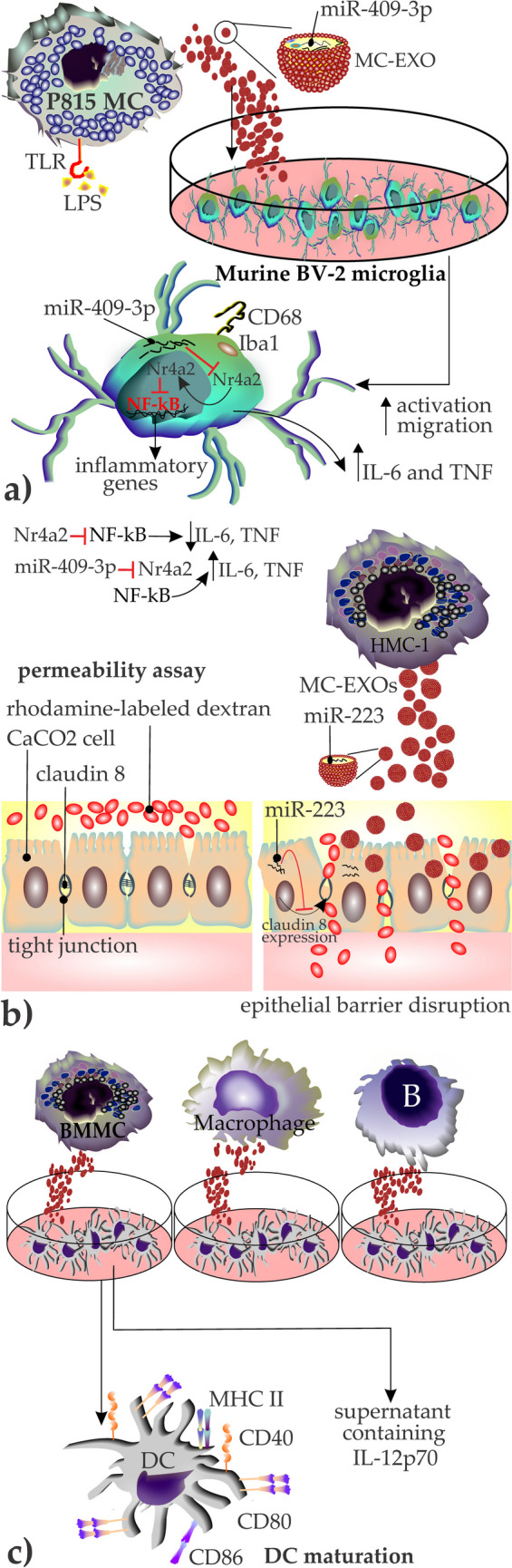
**a** Role of exosomal miR-409-3p in induction of neuroinflammation. MC-EXOs collected from LPS-activated P815 MCs containing miR-409-3p are taken up by murine BV-2 microglia cells and render the cells activated and enhance their invasiveness and migration. At a molecular level, miR-409-3p suppresses Nr4a2 the molecule which prevents the activation of NF-κB pathway. Therefore, miR-409-3p containing MC-EXOs induce the production of inflammatory cytokines including IL-6 and TNF, and support neuroinflammation, **b** HMC-1 EXOs containing miR-223 disrupt epithelial barrier integrity by interfering with the production of tight junction molecules. The schematic representation of a permeability assay in which normally, rhodamine-labeled dextran probes barely can pass through the epithelial cell barrier owing to the effective function of tight junctions. After adding MC-EXOs containing miR-223, the microRNA interferes with the production of claudin 8, an important element of tight junctions, and interrupts the barrier integrity resulting in the passing of the probes from above to the bottom, c) the effect of MC-EXOs on DC maturation and polarization of Th1 cells. Only MC-EXOs among other studied immune cells’ EXOs induced the maturation of DCs and upregulated the markers, especially CD40 and CD80. Mature DCs produce IL-12p70 which is known to contribute to the polarization to the Th1 subset

MC-EXOs carry both mRNA and ncRNA, including microRNAs (miRNAs). These miRNAs commonly bind to their target mRNAs, resulting in translational inhibition or mRNA degradation [75, 76] (Table 2). As of now, little is known about what drives the composition of MC-EXO cargo RNAs. In one study, miR-409-3p was increased in murine P815 MCs activated by lipopolysaccharide (LPS) and in their EXOs. Once murine microglial BV-2 cells were incubated with LPS-P815 EXOs, they showed an increase of miR-409-3p which promoted their activation (by assessment of ionized calcium-binding adapter molecule 1 (Iba1) and CD68 expression) and migration [77]. Additionally, the activated cells had higher releasing levels of inflammatory cytokines IL-6 and TNF. Further molecular investigation uncovered the mechanism, and it was shown that miR-409-3p targets Nr4a2. The latter molecule has been reported to suppress NF-κB [78]. Therefore, miR-409-3p suppresses Nr4a2 through which contributes to the activation of the NF-κB pathway and the production of inflammatory cytokines [77] (Fig. 2a).

**Table 2:** A summary of the characterized microRNAs in MC-EXOs. Additionally, the significance of several of these microRNAs—evaluated directly in serum or cell culture media supernatants, independent of their association with EXOs—is discussed in relation to the pathogenesis of various diseases

MC source	MC type	microRNA(s)	Ref(s)
Mouse	P815	miR-409-3p, miR-6240, miR-3069-3p, miR-5100, miR-7234-5p, miR-470-5p, miR-5619-5p, miR-7647-3p, miR-6979-3p, miR-7022-5p, miR-6973a-5p, miR-3065-5p, miR-5709-3p, miR7082-3p, miR-7221-5p, miR-532-5p, miR-6377, miR-1947-5p, miR-700-5p, miR-3109-5p, miR-499-3p	[77]
human	HMC-1	miR-223, hsa-miR-451, hsa-miR-503, miRPlus-27560, miRPlus-2843, miRPlus-27564, hsa-miR-583, miRPlus-1795, miRPlus-17890, hsa-miR-663, hsa-miR-30b, miR-490, hsa-miR-1226-5p, hsa-miR-4773, hsa-miR-490-5p, hsa-miR-1539, hsa-miR-148a-3p, hsa-miR-17-3p, hsa-miR-7-5p, hsa-miR-140-5p, hsa-miR-625-5p, hsa-miR-590-5p, hsa-miR-296-5p, hsa-miR-181b-5p, hsa-miR-132-3p, hsa-miR-23a-5p	[79, 81, 82]
human	placental MCs	miR-181a-5p	[66]
human	Neoplastic bone marrow residing MCs	miR-21, miR-23-a, miR-25, miR-93, miR-30a	[83]
microRNA		Significance and biofunction	Ref(s)
miR-223		• Its abnormal expression is associated with infectious diseases such as hepatitis, HIV-1, and tuberculosis, disrupting normal processes like neutrophil infiltration, macrophage function, dendritic cell maturation, and inflammasome activation	[84]
hsa-miR-451		• Involved in the regulation of immune cells like T cells, B cells, and neutrophils • Modulation of macrophage M2 polarization	[85, 86]
hsa-miR-503		• Dysregulated in several cancers, like hepatocellular carcinoma. It inhibits proliferation and increases drug sensitivity in tumor cells • Regulates gene expression in several pathological processes, like carcinogenesis, angiogenesis, and tissue fibrosis	[87, 88]
miR-148a		• Promotes apoptosis in colorectal cancer cells by targeting Bcl-2 and activating the intrinsic apoptosis pathway	[89]
miR-490-5p miR-490-3p		• Overexpressed and have higher circulation levels in individuals with breast cancer • A notable negative correlation is reported between their overexpression and a reduction in levels of CD3d, IL-2, and IL-2RA	[90]
miR-17-3p		• Inhibits the activity of phosphatase and tensin homolog (PTEN) through which attenuates myocardial ischemia/reperfusion injury • Protects against excessive posthypoxic autophagy in H9C2 cardiomyocytes via PTEN-Akt-mTOR signaling pathway	[91]
miR-23a-5p		• Acts as a negative regulator of receptor for advanced glycation end-products (RAGE) and downstream activation of ROS signaling, and inhibit COPD progression in vitro and in vivo	[92]
miR-21 miR-30a		• miR-21 and miR-30a have elevated expression levels in individuals with focal segmental glomerulosclerosis whereas miR-21 levels are reduced in individuals with diabetic kidney disease and therefore, may be used as biomarkers in distinguishing these pathologies	[93]

*Bcl*-*2* B cell lymphoma 2, chronic obstructive pulmonary disease (COPD)

MC-EXOs have been reported to disrupt the epithelial barrier. To provide a scientific background for this interaction, Li et al. isolated EXOs from HMC-1 cells, labeled them with PKH67, and separately co-cultured them with various cell lines, including normal cells (NCM460) and colorectal adenocarcinoma cell lines (HT-29 and CaCO_2_). They performed a permeability assay by measuring the traversing of rhodamine-labeled dextran probes through CaCO_2_ monolayers growing on 0.4-mm filters and found that treatment of the intestinal barrier with HMCs-1-EXOs induced the passage of the probes to the bottom. The optical density observed was nearly 4 times more than that obtained from the PBS group as the control. They investigated the molecular basis of this observation and found that HMC-1-EXOs containing miR-223 were taken up by HT-29 and NCM460. To confirm the role of miR-223, they used an inhibition strategy in HT-29 cells by applying miR-223 inhibitor and showed that disruption of the epithelial barrier was significantly reversed once miR-223 was inhibited. This strategy also showed that miR-223 inhibits claudin 8 expression [79] (Fig. 2b). The latter molecule is a member of tight junctions, which is required for a paracellular barrier, and the decrease in its expression results in the collapse of the tight junction barrier [80].

## MC-EXOs Can Modulate Immune Responses

EXOs play a crucial role in modulating immune responses through intercellular communication. These extracellular vesicles can transport functional microRNAs, proteins, and other molecules between cells, influencing both innate and adaptive immunity. For this purpose, they use different mechanisms, mainly through the regulation of activation, expansion, and differentiation [94, 95]. We discuss in this section how MC-EXOs, much like other EXOs, help regulate immune responses in various cell types.

### MC-Derived EXOs Modulate the Immune Response in Cells of the Innate Immunity

#### DC

Dendritic cells (DCs) are professional antigen-presenting cells (APCs) that prime naïve CD4 and CD8 T cells by presenting them with processed antigens [96, 97]. MC-EXOs have been reported to promote the maturation of DCs. In one study, MC-EXO-treated DCs upregulated MHC II, CD80, CD86, and CD40 (particularly CD80 and CD40) [60]. MC-EXOs also upregulated DC production and release of IL-12p70, which promotes Th1 polarization [60] (Fig. 2c).

Importantly, MC-EXOs can modulate DC functions in vivo. Mice injected with OVA-containing MC-EXOs exhibited an immune response in various organs and their resident DC populations. Lymph nodes and spleens were collected 14 h post-injection for analysis. In a parallel setting, they also injected other groups of mice with OVA in the presence of LPS, or with PBS as a control. To investigate the uptake of the OVA-containing MC- EXOs by DCs and their potency of activating T cells, they cocultured DCs with OVA-specific T cell hybridoma 3DO-54.8 and measured IL-2 production by T cells. The results of this step showed that as expected, DCs exposed to OVA-containing MC-EXOs could induce the production of IL-2 in cocultured T cells [60].

#### Mast Cell

MC-EXOs are transferable among different types of MCs isolated from organs or cell lines. In one study, the labeled RNA in MC/9 EXOs was shown to be transferable to other MC/9 cells and HMC-1 cells, but not to CD4 T cells. Interestingly, when HMC-1 cells were exposed to EXOs derived from mouse cell line MC/9, several distinct mouse proteins including CDC6 (O89033), mouse zinc finger protein 271 (P15620), and mouse CX7A2 were identified in HMC-1 that were not present in MC/9-EXOs suggesting that their mRNA may be delivered via EXOs and then translated in HMC-1 cells [67].

### MC-Derived EXOs Modulate the Immune Response in Cells of Adaptive Immunity

**Fig. 3:**
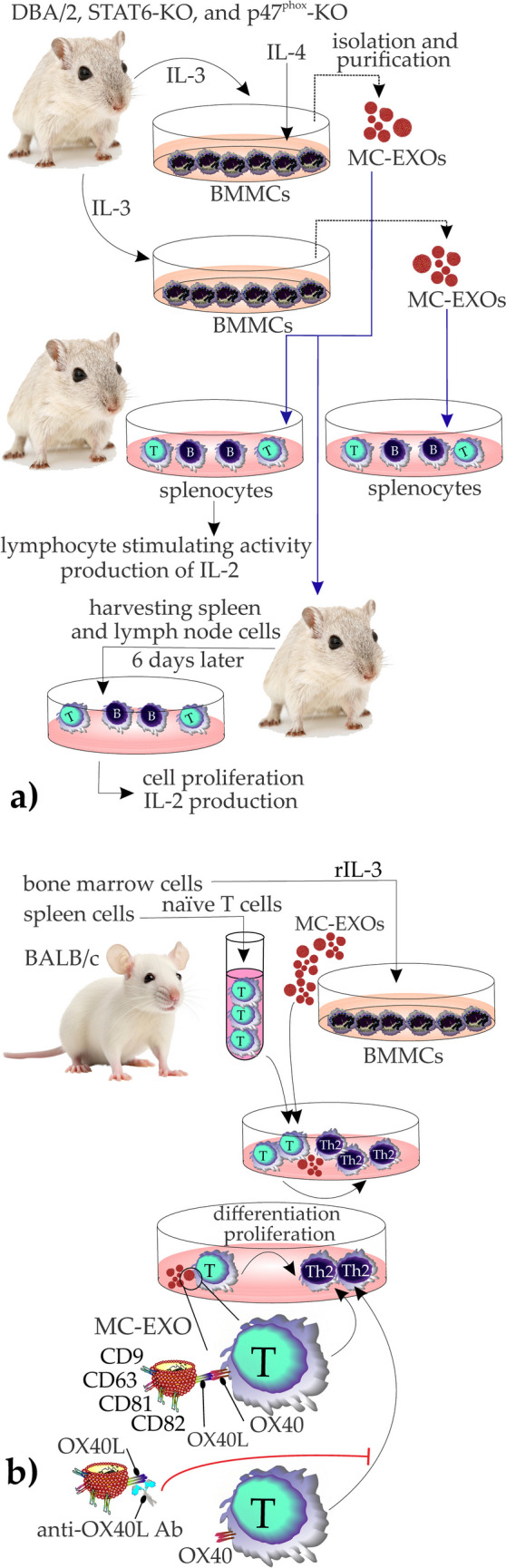
**a** The effects of MC-EXOs collected from mouse cell lines and BMMC on DBA/2 mouse splenocytes. MC-EXOs collected from cultures with IL-4 pretreatment showed efficiency in the induction of lymphocyte stimulatory activity and cytokine production in splenic cells. **b** MC-EXOs interaction with naïve T cells based on OX40L/OX40 axis. MC-EXOs collected from BMMCs were added to naïve T cells. MC-EXOs were not taken up by the cells, instead were shown to have a direct interaction that induced cell proliferation and differentiation into Th2 cells. This effect was reversed once anti-OX40L Ab was applied

BMMCs and MC lines, including P815 and MC9, can induce antigen-independent activation of B and T lymphocytes, a process that does not necessarily require direct cell-to-cell interaction, and the supernatant of MCs exerts their biofunction [68]. The effects of mouse cell lines P815 and MC/9, as well as mouse (DBA/2, STAT6-KO, and p47phox-KO) BMMC-derived EXOs on DBA/2 mouse splenocytes (T and B cells), were investigated by Skokos et al. To study the effects of MCs on the activation of lymphocytes, they cultured mouse BMMC for 19 days in the presence of IL-3 and then treated with combinations of cytokines like IL-4 and IFN-γ for 48 h and cultured them with B and T cells. IL-4 was the only cytokine that primed BMMCs to induce lymphocyte-stimulating activity. The EXOs were isolated from supernatant from 48 h cultures of IL-4-treated BMMC and the EXO-rich fraction could induce both blast formation and proliferation of splenocytes comparable to the initial unfractionated supernatant [68]. A similar effect was shown when P815 and MC/9-derived EXO-rich fractions were studied. However, unlike BMMCs, IL-4 pretreatment was not required to induce exosome production in P815 and MC/9 cells. Interestingly, the investigation of cytokine production showed that the splenic cells treated with BMMCs produced IL-2 and IFN-γ (Th1-type response-associated cytokine) when treated with purified EXOs. In the final step, they injected the mice with BMMC-derived EXOs, harvested spleen, and lymph node cells 6 days later, cultured them for 48 h, and then assessed lymphocyte activation. The results showed that mice injected with EXOs derived from IL-4-treated BMMCs displayed cell proliferation and IL-2 and IFN-γ production in harvested cells. Finally, they studied the EXO-containing molecules and concluded that a phosphatase (CDC25) may exert the lymphocyte activating effect [68] (Fig. 3a).

In another study, BALB/c mice were used to isolate naïve T cells from the spleen. MC-EXOs were then isolated from BMMCs and cocultured with naïve T cells in the presence of IL-4. The results showed that the MC- EXOs induced the differentiation of naive CD4^+^ T cells to Th2 cells as more Th2 cells could be detected in MC-EXO-treated CD4 T cells (almost 28% of cells were IL-4^+^Th2 in the EXO-treated group compared to 13% in the control group, *p* < 0.05). To study the EXOs-T CD4 cells interaction, they used fluorescent dyes Dil and Dio to stain MC-EXOs and T cells respectively and found no sign of MC-EXOs uptake by T cells instead, they adhered to the surface of T cells suggesting a direct cell-to-cell interaction [46]. The authors could confirm the expression of OX40L in both BMMCs and their derived EXOs and concluded that binding of OX40L harboring MC-EXOs to naïve CD4 T cells expressing OX40 primes their differentiation to Th2 cells. This effect could be suppressed using anti-mouse OX40L mAb targeting MC-EXOs. Additionally, peritoneal LNs were collected from healthy BALB/c mice and then CD4 and tryptase were stained to visualize T cells and MCs respectively. They found MCs colocalize closely to CD4 T cells suggesting the existence of an interplay [46] (Fig. 3b).

### Interaction of MCs with Non-Immune Cells Through MC-EXOs

#### Fibroblasts

Fibroblasts are predominant producers of extracellular matrix (ECM) components like structural proteins and adhesive proteins [98, 99]. A well-studied MC-fibroblast crosstalk is based on MC-produced chymase that induces the expression of transforming growth factor-β (TGF-β) which in turn stimulates fibroblast proliferation and collagen production [100]. To determine the effects of MC-EXOs on the function of fibroblasts, Savage and colleagues isolated human lung fibroblasts from COVID-19 patients and exposed them to HMC-1-EXOs. Their results showed that Green (PKH-67)-labeled MC-EXOs were visualized in the cytosol of fibroblasts suggesting that they were actively taken by fibroblasts. Moreover, fibroblasts, treated for 72 h with either MC-EXOs or TGF-β showed comparable levels of collagen secretion and proline hydroxylation [39].

Activation of MCs and induction of MC-EXOs release may induce the production of collagen in fibroblasts which can lead to the progression of fibrosis. Additionally, in the pathogenesis of COVID-19 infection, pulmonary fibrosis and activation of MCs were frequently documented [101–105]. In conclusion, MC-EXOs may play a role in induction of fibrosis by induction of proliferation and ECM component production of fibroblasts.

### Trophoblast Cells

miR-181a-5p is associated with adverse outcomes in patients with preeclampsia [106, 107]. A recent study reported that EXOs derived from placental MCs containing miR-181a-5p were transported to trophoblastic cell line HTR-8/SVneo and regulated their viability, migration, and invasion. Additionally, to assess whether these effects were MiR-181a-5p-dependent, a MiR-181a-5p mimic strategy was applied and showed that MiR-181a-5p mimic further enhanced the inhibitory effect of EXOs, while miR-181a-5p inhibitor attenuated the effect of EXOs suggesting that MiR-181a-5p is the active component of placental MCs-derived EXOs influencing the biofunction of trophoblastic cell line HTR-8/SVneo likely through Yin Yang 1 (YY1)/MMP-9 axis [66]. From a mechanistic point of view, MC-EXOs repressed the expressions of YY1, N-cadherin, Vimentin, and MMP-9 proteins while inducing the expression of E-cadherin protein [66].

### Epithelial Cells

Mast cells coexist closely with epithelial cells in the airways [108, 109]. The reversible process known as the “epithelial-to-mesenchymal transition” (EMT) causes epithelial cells to lose their ability to adhere to one another and to be polar. Instead, they acquire the ability to migrate and invade, transform into mesenchymal stem cells having the capacity to differentiate into a wide range of cell types [110, 111]. Additionally, tissue remodeling, increased MMPs activity, and generation of fibrotic lesions are commonly observed in the process [112–114]. A group of researchers focused on the role of MC-derived EVs in EMT. They obtained EVs from HMC-1 cells and labeled them with the PKH67. A549 cells were exposed to these EVs and the conditioned medium was collected and separated on gelatin-contacting zymogram gels to assess the activity of MMPs. Their results showed that (a) HMC-1-EVs were taken up by A549 cells; (b) after being exposed to HMC-1-EVs, A549 cells’ morphology altered, and they were seen to have elongated protrusions; (c) the expression of several genes involved in EMT including TGFB1, TWIST1 MMP9, and BMP7 was upregulated; (d) E-cadherin protein expression was downregulated while Slug-Snail and MMPs were upregulated; (e) the production of TGF-β, a crucial cytokine involved in EMT, was induced in A549 cells; and (f) MMP-2 and MMP-9 activity in A549 cells’ medium after EV exposure was increased [115].

### Endothelial Cell

Plasminogen activator inhibitor-1 (PAI-1) deactivates both tissue-type plasminogen activator (tPA) and urinary-type plasminogen activator (uPA) [116, 117]. The involvement of MC-EXOs in MC-Endothelial interplay was investigated by Al-Nedawi and colleagues. For this purpose, they collected EXOs from HMC-1 cultures and their protein content underwent a separation step by 2D-PAGE, and protein spots were characterized by microsequencing. Then they incubated these proteins with primary human umbilical vein endothelial cells (HUVEC) and immortalized endothelial cell line EA. hy926 to assess the PAI-1 production and secretion. The results showed that PAI-1 and α1-acid glycoprotein (a PAI-1 activity stabilizer) mRNA expression was increased. Additionally, in response to the exosomal proteins, there was a dose-dependent increase in PAI-1 synthesis. They identified several proteins, including TNF precursor, angiotensinogen, and prothrombinase complex components, including factor V and prothrombin, as the active components after carefully separating the MC-EXOs proteins, testing them independently on endothelial cells, and using proteomic analysis to identify the proteins responsible for the induction of PAI-1 [70]. To conclude, MC-EXOs upregulate PAI-1 secretion from endothelial cells which is a feature of endothelial cell dysfunction and procoagulant states. Mechanistically, MC-EXOs contain TNF precursor which is converted into active TNF. This proinflammatory cytokine is reported to induce PAI-1 in endothelial cells. Furthermore, MC-EXOs transfer substantial amounts of angiotensinogen, which is converted into angiotensin II. This molecule acts as a vasoactive peptide and induces PAI-1 expression [70].

Interestingly, previous studies have highlighted the pivotal role of PAI-1 in mediating interactions between MCs and other cell types. Consistent with these findings, PAI-1 upregulates intercellular adhesion molecule-1 (ICAM-1) receptors on fibroblasts, facilitating their adhesion via integrins expressed on MCs. This binding triggers the activation of both cell types. Upon activation, MCs degranulate, releasing mediators such as histamine, tryptase, and chymase, which in turn activate fibroblasts. Activated fibroblasts exhibit increased expression of α-smooth muscle actin (α-SMA), a marker associated with tissue fibrosis [118, 119].

## Significance of MC-Derived EXOs in the Pathology of MC-Associated Diseases and MC Disorders

The role and significance of mast cells (MCs) in the pathology of allergic conditions, MC-associated diseases, and MC disorders have been extensively studied, with particular focus on their accumulation, activation, and degranulation in the tissues of target organs [120, 121]. This section examines a comparative analysis of MC-EXOs from individuals with these diseases and healthy controls, specifically investigating their production, molecular cargo, and, most importantly, their ability to induce immune responses in other cells.

### Mastocytosis

Systemic mastocytosis (SM) is defined by excessive MC accumulation in tissues because of a somatic gain-of-function mutation in the KIT gene, which hinders MC normal apoptosis and induces MC proliferation [122, 123]. Bones and liver are known to be frequently affected in patients with SM [124–127]. Kim et al. investigated if EVs isolated from patients with SM play a role in osteogenesis, as well as liver pathology [44, 60].

To do so, they used the human cord blood-derived osteoblastic cell line (hFOB1.19) which further differentiates into mature osteoblasts and EVs isolated from serum or bone marrow (BM) of individuals with indolent form of SM. Osteoblast differentiation and maturation were monitored by assessment of the released alkaline phosphatase (ALP), morphologic change into spindled-shaped cells, deposition of extracellular calcium phosphate nodules, expression of RUNX2, and production of osteopontin (OPN) and type I collagen (COL1). In the mouse model, SM-EVs were injected into recipient mice for 2–3 weeks and BM cells were further isolated from femurs and tibias [83].

The following key findings important for bone formation have been identified: (a) patients with SM had higher concentrations of EVs in serum, when compared to healthy volunteers; (b) SM-EV-treated cells show reduced osteoblast maturation, confirmed by reduced ALP activity in cell cultures exposed to SM-EVs, as well as over 50% reduction of calcium deposition, decreased expression of osteoblast differentiation marker RUNX2, decreased production of OPN and collagen type I (COL1), and decreased phosphorylation of AKT and ERK1/2; (c) Annexin 1, CD9, CD63, and ALIX were found to act as the active EV components capable of inhibiting osteoblast differentiation; (d) bone modifying miRNAs, such as miR-21, miR-23-a, miR-25, miR-93, and miR-30a, were upregulated in SM-EVs, and two of them, i.e., miR-23a and miR-30a, were responsible of inhibiting osteoblast differentiation; (e) BM cells from the mice treated with SM-EVs showed a notable decrease in mRNA critical for osteoblast differentiation, such ALP, RUNX2, SMAD1/5, and to a lesser extent SMAD2; (f) long-term injection of SM-EVs to mice reduced bone mass density in long bones [83].

The same team investigated the role of SM-EVs in liver pathology. They used a variety of cells including LX-2 cells (immortalized Hepatic stellate cell line), HepG2 (human hepatoma cell line [128]), and primary mouse hepatocytes, and then exposed them to SM-EVs to monitor the EXO uptake. Additionally, DiD-labeled SM-EVs were injected multiple times into C57BL/6 mice, which were sacrificed 24 h after the final injection. Livers were then collected to prepare slides for histological staining. α-SMA staining was applied as α-SMA acts as an activation and differentiation marker of HCSs into myofibroblasts [73].

The following key identifying the role of EVs in liver pathology have been defined: (a) concentration of SM-EVs correlated with serum tryptase levels in patients with SM; (b) SM-derived EVs induced proliferation and differentiation of HSCs into a fibrotic phenotype with increased levels of α-SMA production, and the expression of tissue inhibitor of metalloproteinases 1 (TIMP-1), COL1, and TGF-β; (c) treatment of HSCs with SM-derived EVs induced the synthesis of proinflammatory cytokines in HSC line, such as IL-6, IL-8, TNF, and monocyte chemoattractant protein 1 (MCP-1); (d) hepatic lysates prepared from livers of mice treated with SM-EVs showed increased levels of α-SMA and human KIT [73].

### Urticaria

Recurrent wheals lasting longer than six weeks without a known etiology are the hallmarks of chronic spontaneous urticaria (CSU) [129–131]. EXOs isolated from the plasma of healthy volunteers (EXs-nor) and CSU patients without antihistamine sensitivity (EXs-CSU-S) or resistance (EXs-CSU-R) were extracted and incubated with HMC-1 in a study conducted by Fang et al. The results demonstrated that CSU-EXs derived from the patients have significantly increased tryptase-1 mRNA expression, as well as tryptase and histamine levels, and the expression of TLR-2, TLR-4 (both at mRNA and protein levels), and phosphorylation of MAPK in HMC-1 cells. Overall, the effects were more notable once HMC-1 cells were treated with EXOs isolated from the group resistant to treatment with antihistamines. The researchers assessed the production of inflammatory cytokines including IL-6, TNF, IL-4, VEGF, CXCL-1, CXCL-5, and CCl-2 in cell supernatants and the results portrayed a significant elevation in the levels of these cytokines in CSU patients compared to healthy controls. The levels of these cytokines were also higher in the supernatant of HMC-1 cells treated with EXs-CSU-R [132]. Application of TLR2, TLR4, and MAPK inhibitors provided insight into the mechanism of action as applying this strategy reduced the levels of inflammatory mediators. The two main limitations of this study were not determining the cell source of EXOs by defining the signature markers and not providing the ligand of studied TLRs or any other serum component(s) capable of MC activation [132].

### Asthma

MCs are frequently observed to accumulate and degranulate in the lungs of individuals with asthma, playing a pivotal role in the disease’s pathogenesis. Through the release of various mediators, MCs contribute to key pathological processes, including (a) bronchoconstriction (like histamine and LTC4) [133], (b) airway hyperresponsiveness (such as tryptase) [36], (c) airway remodeling (including IL-4, basic fibroblast growth factor-2 (bFGF-2), and tryptase), and (d) mucus hypersecretion (like LTC4 and IL-13) [36].

The previously published paper of Almqvist et al. contains interesting results in brief, allergy sensitization in naive recipient mice was alleviated by intraperitoneal injection of isolated serum or serum EXOs collected from tolerized OVA-fed BALB/c mice. In the mice receiving serum EXOs from OVA-fed donors, the IgE levels were notably lowered [134]. These results inspired Xie and colleagues to verify these results in the context of asthma. They postulated that FcεRI on the surface of released EXOs may capture free IgE.

They isolated MC-EXOs from BMMCs generated from BALB/c and FcεRIa^−/−^ BALB/c mice controls. Both types of MC-EXOs were then incubated with IgE-DNP and then added into BMMC suspensions. After 1 h of incubation, BMMCs were checked by flow cytometry using anti-IgE-PE. The results showed that the fluorescence of BMMCs was inversely proportional to the dose of BMMC-EXOs preincubation with IgE suggesting that IgE is being captured by FcεRI on EXOs. They also designed a mouse allergic asthma model using OVA. Then, 30 min before the challenge of OVA inhalation, BMMC-EXOs were injected intravenously into mice and this process was repeated once a week [72]. Moreover, they measured the levels of histamine, IL-4, IL-5, IL-10, IL-13, and IFN-γ in the supernatants of bronchoalveolar lavage fluid (BALF), and serum OVA-specific IgE levels. The airway hyperresponsiveness (AHR) of OVA-treated mice was alleviated over time with the administration of BMMC-EXOs, which was accompanied by a reduction in OVA-specific IgE in serum and histamine levels in BALF [72, 135]. Application of MC-EXOs reversed the rise in Th2 cytokines (IL-4, IL-5, and IL-13) levels, and the decrease in Th1 cytokines (IL-10 and INF-γ) induced by OVA challenge. Overall, their study showed that MC-EXOs bind to free IgE via FcεRI, therefore depleting IgE essential for MC activation [72].

## Significance of MC-Derived EXOs in the Pathology of Non-Allergic Diseases

The significance of MC-EXOs in transporting bioactive molecules that influence physiological and pathological processes in tumors has been studied using corresponding tumor cell lines. This section explores how MC-EXOs affect characteristics such as invasiveness and migration in recipient cells. Additionally, we highlight the impact of their cargo on the recipient cells’ secretome, surface marker expression, and ability to regulate or shape immune responses.

### Atherosclerosis

We recently reviewed the role of MCs in the progression and destabilization of atherosclerotic plaques ending in their rupture [136]. MCs have been widely reported to accumulate in anatomic sites of plaques. IgE-dependent activation of plaque-residing MCs may result in their degranulation and release of proinflammatory mediators and proteases such as MMPs that degrade ECM components and make the plaque susceptible to rupture [137, 138].

Yang et al. used OVA-sensitized ApoE^−/−^ mice to develop chronic asthma to find a link between asthma and atherosclerosis. Additionally, they used ApoE^−/−^FcɛR1α^−/−^ mice and ApoE^−/−^ FcɛR1α^+/+^ (WT) as control mice to focus on the role of IgE and its corresponding signaling via FcɛR1. Furthermore, they applied omalizumab (a humanized monoclonal antibody that selectively binds to circulating IgE, thus preventing MC activation [139, 140]), cromolyn (a MCs stabilizer or inhibitor), and dexamethasone to check if their antiallergic properties work also for asthma-induced atherosclerosis. They grouped the mice as follows: WT mice treated with PBS, KO mice treated with PBS, WT mice treated with OVA, and KO mice treated with OVA. From day 21 onward, mice were exposed to aerosolized OVA or PBS according to their grouping strategy. They also isolated EXOs from LAD-2 cells. We summarized the main findings as follows:

(a) Compared to the PBS-WT mice, OVA-sensitized WT asthma mice showed significantly more atherosclerotic lesions and larger atherosclerotic lesions in the aortic root. These findings were alleviated in OVA-sensitized ApoE^−/−^FcɛR1α^−/−^ mice suggesting a role for IgE-FcεR1 pathway; (b) omalizumab could effectively alleviate asthma-induced atherosclerosis by blockade of IgE highlighting the role of IgE and its signaling in the studied pathology; (c) dexamethasone or cromolyn could partially inhibit asthma-induced atherosclerosis and inflammation in vivo, suggesting the involvement of another mechanism in asthma-induced atherosclerosis; (d) PKH67-labeled EXOs derived from P815 cells injected into C57BL/6 J mice were taken into the vascular endothelium and cerebellar degeneration-related protein 1 antisense RNA (CDR1as) was increased in the vascular endothelium; (e) EXOs from IgE-activated LAD2 cells showed an upregulation of circRNA CDR1as; (f) intercellular adhesion molecule-1 (ICAM-1) and vascular cell adhesion molecule-1 (VCAM-1) were substantially upregulated in endothelial cells after treatment with MC-EXOs. They concluded that MC-EXOs from IgE-activated MCs carrying CDR1as may affect endothelial function and support the aggravation of atherosclerosis [141].

### Lung Adenocarcinoma

MCs can promote tumor growth in various adenocarcinomas through multiple mechanisms, including enhancing the tumor's vascular supply, facilitating proteinase-mediated ECM degradation, and inducing immunosuppression. Upon activation, MCs release a range of angiogenic mediators and cytokines, such as VEGF, FGF-2, tryptase, chymase, IL-8, TGF-β, TNF, and nerve growth factor (NGF) [142].

PKH67 dye-labeled HMC-1-EXOs were reported to be taken up by A549 cells and induced the proliferation of A549 cells which was assessed by BrdU. Additionally, after seeding A549 cells on the membrane of the lower chamber with various doses of HMC-1-EXOs present in the upper chamber, it was shown that more A549 cells migrated into the upper chamber in a dose–response dependent manner suggesting a role for HMC-1-derived EXOs in induction of migration in lung adenocarcinoma cells. MC-EXOs in this experiment were carrying KIT protein, therefore, the next question for which the team went a step further was “What is the consequence of delivering this KIT-rich cargo to A549 cells”? They observed an enhanced proliferation of MC-EXOs-treated A549 cells. They found an increased phosphorylation in PI3K, AKT, and glycogen synthase kinase-3 β (GSK3β). To have a better vision of the link between the phosphorylation of these molecules and the proliferation of cells, one should note that phosphorylated PI3K phosphorylates AKT which in turn inactivates GSK3β by phosphorylation. Cyclin D1 is a cell-cycle regulator downstream of GSK3β inhibited by GSK3β. Deactivation of GSK3β results in a continuing cell cycle from the G1 phase into the S phase (proliferation) [64].

### Hepatocellular Carcinoma

The interaction between MCs and tumor cells of different types is based on direct cell-to-cell or cytokine-based interaction and surely is bidirectional. Recent works magnified the role of MC-EXOs in tumor immunology. Xiong et al. focused on the exosome-based interaction of MC/tumor cells and reported important findings. EXOs were collected from the supernatant of HCV-E2-stimulated HMC-1 cells, labeled with PKH67, and incubated with HepG2 for 24 h, and miRNA microarray assay and western blot were conducted on stimulated or unstimulated HMC-1 cells. Their labeling strategy helped them confirm that Alix and CD63-positive MC-EXOs were taken up by HepG2 cells. A critical feature of the development of cancer is tumor cell migration, which is especially significant during invasion, the first stage of metastasis [82, 143, 144], the team aimed to investigate this concept in their study therefore, incubated HepG2 and Hep3B cells with PBS, normal MC-EXOs, and HCV-E2-stimulated MC-EXOs. Their findings showed that the migration and invasion of HCC cells were markedly reduced by HCV E2-stimulated MC-EXOs [82]. They also reported that miR-490 (a widely recognized tumor suppressor miRNA) was upregulated in MC-EXO induced by HCV-E2 stimulation orchestrated the molecular events leading to having a more aggressive phenotype of HepG2 cells [82].

### EXOs from Other Cellular Sources Modulate the Immune Activity and Secretome of MCs

#### Lung Cancer-Derived EXOs

Co-incubation of C57BL/6 mouse BMMCs with A549 lung adenocarcinoma cell line-derived EXOs for 24 h provided evidence of how EXOs influence the secretome in MCs. EXOs induced the degranulation in MCs according to the results of the β-hexosaminidase release test. Additionally, among the cytokines and mediators assessed, IL-13, SerpinE1, thrombopoietin, and CXCL16 showed a significant change when compared to control [145]. However, these macromolecules’ biofunction suggests a connection to coagulopathy. SerpinE1 (also known as plasminogen activator inhibitors or PAI-1) acts as a serine protease capable of inhibiting tissue plasminogen activator (tPA) and urokinase, which are activators of plasminogen and fibrinolysis, respectively [146, 147]. Considering that MCs may be linked to lung cancer-related thrombosis by releasing these mediators, the role of tumor-derived EXOs in the activation of MCs and regulating their secretome gains significance.

In another study, labeled A549-derived EXOs were co-incubated with mouse BMMCs and showed that the BMMCs uptake EXOs. The increase in β-hexosaminidase release proved that MC became degranulated and the assessment of the MC-secretome showed an increase in the levels of TNF, MMP-9, tryptase, and IL-6 in the supernatant of treated MCs. The research team, in the next step, collected the supernatant and added it to HUVEC cells in the presence of CCK-8 cells to evaluate the proliferation. The results showed that the supernatant could significantly induce the HUVEC cells' proliferation and migration. This effect was due to the tryptase released from MCs which acts through protease-activated receptor 2 (PAR-2) (mechanistically this binding results in upregulating the phosphorylation of JAK and STAT in HUVEC cells) [148].

### Extracellular Vesicles from Packed Red Blood Cells

Constant depletion of ATP resources in donated packed blood units affects the normal function of RBCs’ membrane such as the decrease in the activity of plasma membrane Ca^2+^ pumps leading to boosting RBC calcium and dysregulating the normal function of the enzymes like flippase, floppase, and scramblase their function maintains the phospholipid asymmetry. Calcium-dependent activation of RBC scramblase results in inhibition of flippase and phosphatidylserine externalization, cytoskeletal proteolytic degradation, and band 3 aggregation, eventually resulting in vesiculation and the release of RBC-EXOs [149, 150].

RBC-derived EXOs have been reported to modulate immune responses in MCs. Fang et al., in their study aimed to answer the following questions, “Are RBC-derived EXOs taken up by MCs, and if so, can they modulate the immune response? And what are the mechanistic pathways involved in influencing MC immunobiology once being exposed to these EXOs?” They collected healthy volunteers’ plasma EXOs (nor-EXOs) and packed red blood cells’ EXOs (RBC-EXOs) and incubated them with HMC-1. They found that RBC-EXOs could induce the expression of tryptase-1 and prostaglandin D2 (PGD2), inflammatory mediators, and the levels of TLR-3 and MAPK in HMC-1 cells. Furthermore, the expression levels of IL-6, TNF, IL-4, INF-γ, CCl-2, CXCL-1, PAF, LTB-4, and VEGF-mRNA were significantly higher in RBC- EXOs treated HMC-1 cells. Application of MAPK inhibitors and a TLR-3/ dsRNA complex inhibitor could reverse the effects of RBC-EXOs on HMC-1 cells suggesting that TLR-3 and MAPK pathways play a role in orchestration of these responses [151].

Table 3 offers a more thorough summary of the impacts of EXOs and EVs from a range of cells when treated with various MC types.

**Table 3:** A summary of effects of other cells derived EXOs or EVs on MCs

EXOs or EV source cell	Type of MCs	Biologic effects	Ref
**A549 cell line** (human non-small cell lung cancer cell line)	BMMCs of C57BL/6 mouse	• IL-13, SerpinE1, thrombopoietin, and CXCL16 were upregulated	[145]
**A549 cell line**	BMMCs	• TNF, MMP-9, tryptase, and IL-6 were upregulated	[148]
**Human bone marrow-derived MSCs**	Mouse BMMCs LAD-2	• Calcium ionophore A23187-mediated activation of both cell types was suppressed after treatment with MSC-Derived MVs • MCs produced significantly lower levels of TNF • BMMC production of PGE2 was induced after MV exposure • MV exposure could also up-regulate the mRNA expression of COX2, the key enzyme involved in PGE2 synthesis • MCs respond to PGE2 by expressing receptors E-Prostanoid (EP)1–4. MV treatment could increase the expression of EP4 in MCs through which exerts anti-inflammatory effects (decrease in TNF levels)	[152]
**H1299 cell line** (human non-small cell lung carcinoma cell line) **Mia PaCa-2** (pancreatic cancer cell line)	LAD-2	• Phosphorylation of the ERK1/2 MAP kinases in a dose-dependent manner, resulting in MC activation • MC activation was associated with adenosinergic signaling by the adenosine A3 receptor • Upregulation of the expression of angiogenic and tissue remodeling genes, including IL8, IL6, VEGF, and amphiregulin (a ligand of the EGFR)	[153]
**H1299, H1975, A549** (human non-small cell lung cancer cell lines)	LAD-2	• ERK phosphorylation was induced in LAD-2 cells resulting in their activation • Induced MC migration and increased release of cytokines and chemokines, such as TNF and MCP-1/CCL2	[154]
**Activated Jurkat T cells**	LAD-2	• Augment ERK phosphorylation and IL-8 release by delivering miR-4443 and downregulation of expression of Protein Tyrosine Phosphatase Receptor type J (PTPRJ) gene	[155]
**Activated Jurkat T cell lymphoma cells**	Primary cultured human CBMCs and LAD-2	• Microvesicles induced the upregulation of cytokine/cytokine receptors in LAD-2 cells including Inhibin, beta A (fold change: 34.51), CCL7 (fold change: 25.83), CCL4 (fold change: 21.77), IL-24 (fold change: 9.35), etc. Additionally, the MVs induced IL-24 mRNA expression in CBMCs • Microvesicles induced the production of IL-24 in LAD-2 cells • ERK, JNK, and STAT3 play a role in induction of MC production of IL-24	[156]
**Activated Jurkat T cell lymphoma cells**	Primary cultured human CBMCs and LAD-2	• MVs induced the activation of MAPK signaling pathway, degranulation (measured by b-hexosaminidase releasing test), and IL-8 and oncostatin M release from both MC types	[157]
**Tonsil-derived mesenchymal stem cells (T MSCs)**	HMC-1	• Exposure of T-MSC-EXOs influences the transcriptomic changes in HMC-1 cells • TLR3 (poly I:C) or TLR4 (lipopolysaccharide) primed T-MSC-EXOs induced fewer transcriptomic changes than unprimed T-MSC-EXOs in HMC-1	[158]

*CBMCs* cord blood mast cells, *MSCs* mesenchymal stem cells, *BMMCs* bone marrow-derived MCs

## MC-Derived Mediators Can Be Intracellularly Transferred by EXOs from Target Cells

In the above-described sections, we extensively went through the EXO-orchestrated immunologic interaction of MCs with other cells or vice versa [159]; however, a third setting was also reported in which the target cell-derived EXOs after being released uptake MC mediator(s) and then deliver their cargo to the target cell.

**Fig. 4:**
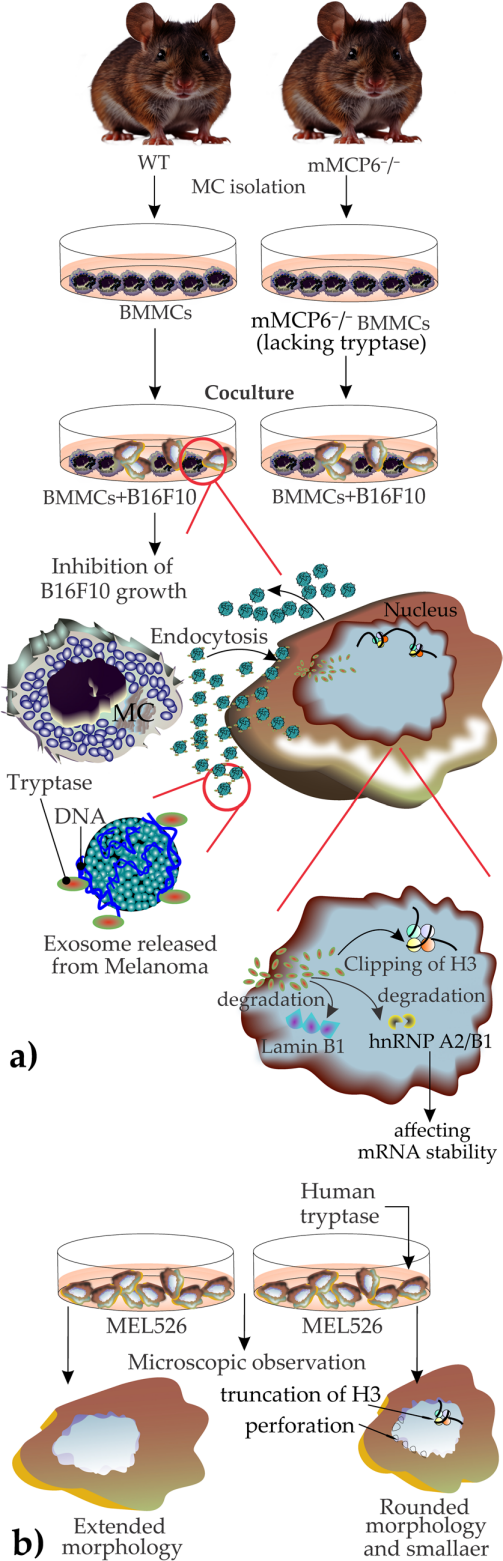
**a** Impact of tryptase on mouse melanoma tumor cells: bone marrow-derived mast cells (BMMCs) were collected from wild-type (WT) and tryptase-deficient (mMCP6^−/−^) mice with a C57BL/6 J genetic background. BMMCs were co-cultured with B16F10 mouse melanoma cells. The growth of melanoma cells was inhibited only when co-cultured with WT BMMCs. The melanoma cells released DNA-coated exosomes that bound to mast cell tryptase. These tryptase-loaded EXOs were subsequently endocytosed by melanoma cells, facilitating nuclear entry of tryptase, which primarily led to the clipping of H3 histone, degradation of Lamin B1, and degradation of heterogeneous nuclear ribonucleoprotein A2/B1 (hnRNP A2/B1), thereby affecting mRNA stability, b) Impact of human tryptase on human melanoma cells (MEL 526): human melanoma cells (MEL 526) were treated with human tryptase and compared to untreated control cells. Similar to mouse melanoma cells, human melanoma cells internalized tryptase, which, upon reaching the nucleus, led to H3 histone truncation, nuclear membrane perforation, and morphological changes. These changes were observed microscopically, showing a shift from an extended morphology to a rounded morphology with reduced cell size

In one example of such a setting, Melo and colleagues focused on the interaction of MC and melanoma cell lines. They isolated BMMCs from wild-type and tryptase-deficient (mMCP6^−/−^) mice and cocultured them separately with B16F10 mouse melanoma cells or MEL526, MM466, and MM253 (human melanoma cells) to decipher the role of MC mediators on different aspects of melanoma cells’ biology and then studied the content of melanoma cell-derived EXOs and their carrying MC mediator content [160]. Their results are briefly summarized in the following:

(1) MCs exert their inhibitory effect on B16F10 cells growth through their released tryptase in which only WT MCs showed such an effect; (2) MC-released enzymatically active tryptase was delivered to the nucleus of mouse melanoma cells where it initiated core histone processing including truncation of histone 3 (H3), degradation of Lamin B1, and degradation of heterogeneous nuclear ribonucleoprotein A2/B1 (hnRNP A2/B1, a molecule involved in mRNA stability); (3) tryptase inhibited the proliferation of the studied melanoma cell lines without inducing apoptosis or necrosis; (4) melanoma cells-derived EXOs coated with DNA uptake the MC-released tryptase and tryptase binds to surface DNA (tryptase, normally is compressed in MC granules while binding to negatively charged heparin, likewise, DNA, which is chemically a linear polyanionic molecule acts similarly [161–163]); and (5) labeled purified EXOs incubated with tryptase were taken up by melanoma cells and were tracked in the cytoplasm and nucleus (6) recombinant human (rh)-tryptase exerted morphological changes on MEL526 cells and shifted them from extended to a rounded morphology (decreasing cell size) [160] (Fig. 4).

## Unmet Questions

In the previous section, we focused on the biological effects of EXOs derived from different types of MCs and the way they collaborate to orchestrate immune responses in different immune and non-immune cells. In this section, we aimed to highlight the remaining knowledge gaps and compiled a list of open questions to inspire further research by our colleagues in the field. Table 4 represents the current knowledge gaps.

**Table 4:** Unmet questions regarding the role of MC-derived EXOs in the orchestration of immune responses

Unanswered questions and the corresponding background	Ref
**Which cytokines induce the production and release of EXOs in different MC types?** According to the literature, while MC lines including P815 and MC/9 release EXOs in the absence of IL-4, mBMMCs need this cytokine to produce EXOs. The effects of other cytokines on the capacity of various MC types, including different cell lines and human tissue-isolated MCs, to produce and release EXOs remain to be investigated	[68]
**Do MC-EXOs released from resting and degranulated MCs differ in their content? If so, can content profiling of EXOs provide further information on the activation of the cells?** MCs release EXOs regardless of their activation status, however, it is poorly understood whether their exosomal cargo differs depending on the activation of MCs. Linking these findings to pathologies driven by mast cell activation through autoimmune mechanisms, such as Type I and IIb autoimmune subtypes of CSU, where skin MCs become activated through FcεRI pathway, could offer a clinical perspective with potential treatment implications	[23, 39]
**Is there a difference in the content of MC-EXOs derived from both main subsets of human MCs?** Human MCs are classified into two main subsets (MC_T_ and MC_TC_), each having a different secretory profile and protease content. Our knowledge regarding the differences between the EXOs contents derived from these two subsets is still rudimentary	[164–166]
**Which MC mediators, released in response to tumor-derived EXOs, may contribute to the progression of tumors? Do tumor-derived EXOs differ in their content during different stages of the tumor?** Tumor microenvironment (TME) is a complex entity in terms of cellularity and its complicated cell-to-cell interactions. The role of MCs in reshaping the tumor microenvironment has been studied. Most recently the involvement of tumor-derived EXOs in the activation of tumor residing MCs was reported. Indeed, this mechanism provides a positive loop in favor of tumor progression in which tumor-derived EXOs activate MCs and induce their degranulation, and MCs in return produce mediators including SerpinE1 which may induce their growth. Elucidating the change in EXO contents of tumor cells in different stages of tumor progression and the effect on MC secretome may shed light on the dynamic of the EXO-based MC-tumor cells interaction	[8, 44, 167, 168]
**How do tumor cell-derived EXOs activate MCs?** The molecular mechanism by which MCs become activated upon EXO exposure remains to be fully elucidated. In one study, it was reported that the stem cell factor (SCF) content of EXOs interacts with KIT. It is yet unknown which other possible ligands in MC-EXOs could activate MCs through binding to MC-expressed receptors	[148]
**Do MC-EXOs carry active MMP-2 and MMP-9?** Gelatinases include Gelatinase A (MMP-2) and Gelatinase B (MMP-9). These zinc-dependent matrix metalloproteinases play a crucial role in the degradation of ECM proteins including collagen IV and V, laminin, and chondroitin sulfate. They are associated with several biological processes including metastasis, ECM turnover, invasiveness of tumor cells, etc. They have been reported in several types of EXOs, however, their presence and role in MC-EXOs have not been investigated	[169–172]
**Does the concentration of MC-EXOs correlate with symptoms or stage of an allergic disease?** During preparation of this review, we studied several papers reporting an increase in the load of released MC-EXOs in allergic disorders, however, it has not been addressed whether the increase in the load of MC-EXOs in serum or other fluids correlates with the disease stage, progression, or the levels of MC mediators. Additionally, the proteomics or genomics comparison among MC-EXOs released in the serum of individuals with an allergic disease but in different stages or types (especially mastocytosis and chronic urticaria) may reveal new aspects of MC-EXOs in understanding the pathology and orchestrated immune responses in allergic diseases	[73, 83]
**What is the link and relevance between the MC production of extracellular traps (ETs) and the release of MC-EXOs?** MCs produce and release ETs once they are exposed to special chemicals or pathogens. The process is accompanied by special chromatin changes, decondensation of chromatin, the release of DNA from the nucleus, attachment of molecules including proteases and molecules with anti-microbial activity (like LL-37), and release of these structures from the cell. It would be interesting to decipher if the production of MC-EVs alters just before the cell death because of MC-extracellular trap formation. And a step further, which fingerprint molecules are altered or remain unaltered in MC-EXOs before and after MCETosis?	[162]
**Do the amounts of released MC-EXOs depend on the MC activation pathway?** MCs become activated through different pathways (IgE: FcεRI, Neuropeptides: MRGPRX2, C5a: C5aR, etc.). It could be interesting to compare the MC-EXOs derived from the same MC type or different MC sources, their markers, and their ability to exert immune responses in other cocultured cells	[26, 141]
**Can routine MC secretagogues induce MC-EXO release? If so, which is the most capable one?** Secretagogues including anti-IgE and anti-FcεRI antibodies, MRGPRX2 activators including compound 48/80(C48/80), SP, cortistatin, and calcium ionophore A23187, ionomycin, and non-ionic detergent triton X-100 as well as cationic peptide Mastoparan 7 activate and prompt MCs to degranulation differently in the context of mechanism and the percentage of beta-hexosaminidase release. It could be interesting to compare their capability in the induction of EXO release on the same (or various) MC type	[173–175]
**What are the main molecules enclosed in different types of MC-EXOs capable of initiating the immune responses in their target cells?** According to Table 1, there are still many types of MCs either cell lines (LUVA and MC/9) or tissue-isolated ones (in particular human organ-derived MCs) their active molecules need to be investigated	[65]
**Are MC-EXOs varying in morphology contain different sets of MC signatures or act differently in shaping immune responses?** MC-EXOs after isolation have been visualized in different shapes, sizes, and arrangements under the microscope and applying negative staining. Zabeo et al. in their intricate study, could identify, quantify, and classify MC-EXOs from a single cell line (HMC-1) in 9 subpopulations such as oval vesicle, small tubule, pleomorphic vesicle, single and small double vesicle. It is worthwhile comparing the morphology of MC-EXOs isolated from different types of MCs, to determine if some of the shapes appear mostly in a specific MC activation pathway and basically, if they carry specific sets of MC signatures	[176]
**Do MC-EXOs derived from various MC types carry MHC-II molecules?** MCs in addition to MHC-I molecules, express MHC-II which enables them to act as APCs. Although the presence of MHC-II molecules (and MHC-II-associated molecules) has been reported in some EXOs of immune cells, MC-EXOs of humans have not been investigated for the expression of these molecules	[177–179]
**Do MC-EXOs play a role in MC-orchestrated host defense against invading pathogens?** The anatomic distribution of MCs in the host-environment interface, including skin, respiratory epithelium, and gastrointestinal tract makes them act as immune sentinel cells. They employ a variety of strategies to eliminate pathogens such as the production of ETs, and the production of Reactive Oxygen Species (ROS). In vitro studies have shown coculturing of MCs with parasites can significantly reduce the recovery and viability of parasites. The role of MC-EXOs in the modulation of physiologic processes such as protein synthesis in pathogens as another strategy of MC can be an exciting field of research when a variety of MC-EXOs derived from human skin or mouse peritoneal MCs coculture with pathogens responsible for diseases	[180, 181]
**Do MC-EXOs modulate the immune response in cells of innate and adaptive immunity in addition to those listed in subsections 3.1 and 3.2?** Looking at these two subsections, one can easily find out that there are many other cells that MC-EXOs may mediate modulation of immune responses, however, have not been thoroughly investigated or we yet have a general and basic knowledge. Colleagues are encouraged to cover this by referring to the strategies, methods, and techniques used for other cells represented in this review (**Sect. 3**). All this applies to EXOs of other cells when coculture with MCs (**Sect. 6**)	[59]
**Can MC-EXOs act as potential therapeutic agents?** In **Sect. 2.2** we addressed a piling up list of miRNAs having different expression levels in between the originating parental MCs and their derived EXOs. Considering that miRNAs interfere with the gene expression in both normal or tumor cells and that many miRNAs have higher expression levels when compared to the originating MCs (plus that they are the smallest EVs (30–150 nm) capable of penetrating cells and tissues and crossing barriers like blood brain barrier (BBB), the idea of their application in tumor progression control comes under the spotlight	[182–185]

## Discussion and Conclusion

Most cells release EXOs, which are primarily involved in the presentation of antigens, the induction of tolerance, and the transfer of genetic material. Different techniques are used to isolate MC-EXOs including microfiltration, precipitation, and ultracentrifugation after which electron microscopy, flow cytometry, and proteomic analysis are widely used to confirm their effective isolation and content [186]. The process of uptake of MC-EXOs by their target cell not only may phenotypically alter the cell, its function, or secretome but even gives it a portion of MC fingerprint molecules their functionality awaits to be explored. As an example, EVs isolated from the serum of patients with SM containing MC-specific markers after being taken up by HSCs, transfer and deliver their MC signature molecules that later become expressed by HSCs [73]. The immunogenicity of antigens associated with EXOs makes them act as entities carrying a cargo of antigens against which different classes of Abs are produced, for instance, EXOs purified from DBA/2mice BMMC cultured with transferrin could induce the production of IgG1 and IgG2a when injected into syngeneic DBA/2 mice [60]. The capacity of EXOs to carry external antigens can make them a useful platform for the induction or suppression of immune responses. In another study, OVA-enriched mesenchymal stem cell (MSC)-derived EXOs were sublingually applied to Balb/c mice and then the mice were sensitized by intraperitoneal injection of OVA. In mice receiving OVA-enriched EXOs, allergic OVA sensitization was largely inhibited and the mice had reduced OVA-specific IgE and IL-4, and elevated TGF-β levels [187]. MC-EXOs may have the potential to carry specific antigens and to be used prophylactically to control allergic disorders.

Despite our partial understanding of the biogenesis, uptake, trafficking, and immunology of MC-EXOs, there remain many more unanswered questions than those we included in our list of unmet question table, which highlights the need for additional MC-EXOs studies. The ability of EXOs to convey different therapeutic agents, like proteins, mRNAs, ncRNAs, and lipids, to particular cell types or tissues has generated significant interest in the use of EXOs for targeted medication delivery. EXOs can traverse the blood–brain barrier, penetrate tissues, and circulate into the bloodstream. Our understanding of MC-EXOs can be expanded by taking these capabilities into account and then applying bioengineering. As an example, rat MC line RBL-2H3 was engineered to express human MHC-II molecules loaded with HLA-DR1 hemagglutinin (HA) acting as the antigen. RBL-2H3 cells were activated to release EXOs (bearing HLA-DR1-HA) capable of partially activating HLA-DR-1-HA specific T cells. Then the researchers improved the efficiency of these EXOs by crosslinking them to latex beads [188]. Bioengineering of MC-EXOs to express MC-specific markers of human or mouse origin will provide the opportunity to expand our knowledge of how MCs communicate and interact with other cells by releasing MC-EXOs. Furthermore, they can be used to understand further aspects of allergic disorders by comparing the contents of MC-EXOs of individuals with MC-dependent diseases mainly urticaria and mastocytosis to healthy individuals.

**Fig. 5:**
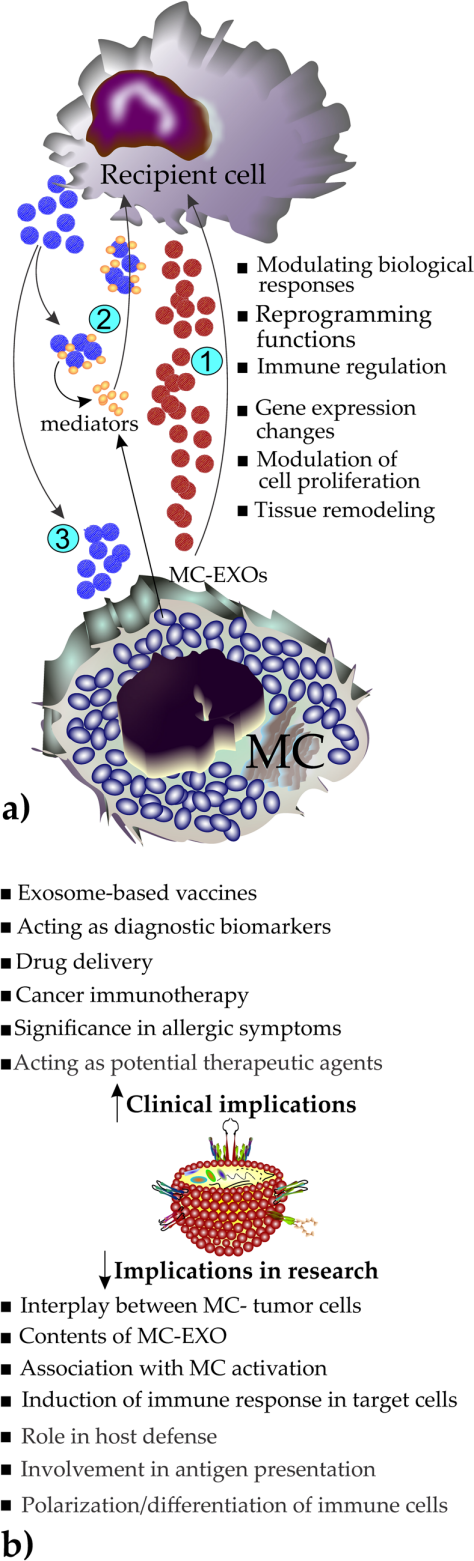
**a** An overview of the EXO-involved mechanisms in mast cell (MC) and recipient cell interactions: (1) MCs release MC-EXOs, which exert various biological functions in the recipient cell, such as altering biological responses, gene expression, and more. (2) The recipient cell releases EXOs, which may capture MC-released mediators, particularly tryptase, before being endocytosed by the recipient cell. (3) The recipient cell releases EXOs that may, in turn, exert biological functions on MCs. **b** A perspective of potential implications of MC-EXOs in research and clinical applications

In MC biology, MC-EXOs are important drivers of intercellular communication that carry a diverse cargo of proteins, lipids, and RNAs that can be delivered to recipient cells, modulating their biological responses and reprogramming cellular functions (Fig. 5a). Understanding the role of MC-EXOs in health and disease, particularly in allergic and non-allergic conditions, opens new avenues for therapeutic interventions targeting EXO-mediated pathways (Fig. 5b).

## Data Availability

No datasets were generated or analysed during the current study.

## Abbreviations

ALIX: ALG-2-interacting protein X
ALP: Alkaline phosphatase
APC: Antigen presenting cell
BALF: Bronchoalveolar lavage fluid
BMMCs: Bone marrow-derived MCs
BSA: Bovine serum albumin
CDC25: Cell division cycle 25
CDR1as: Cerebellar degeneration-related protein 1 antisense RNA
CNS: Central nervous system
CSU: Chronic spontaneous urticaria
ECM: Extracellular matrix
EMT: Epithelial-to-mesenchymal transition
EVs: Extracellular vesicles
EXOs: Exosomes
GSK3β: Glycogen synthase kinase-3 β
HCV-E2: Hepatitis C virus E2 envelope glycoprotein
hnRNP A2/B1: Heterogeneous nuclear ribonucleoprotein A2/B1
HSCs: Hepatic stellate cells
HSP70: Heat shock protein 70
HUVEC: Human umbilical vein endothelial cell
Iba1: Ionized calcium-binding adapter molecule 1
ICAM-1: Intercellular Adhesion Molecule 1
MC-EXOs: Mast cell-Exosomes
miRNA: MicroRNA
MRGPRX2: Mas-related G protein-coupled receptor X2
MSC: Mesenchymall stem cell
MVBs: Multivesicular bodies
ncRNAs: Noncoding RNAs
NGF: Nerve growth factor
Nr4a2: Nuclear receptor subfamily 4 group A member 2
OVA: Ovalbumin
PAI-1: Plasminogen activator inhibitor-1
PGD2: Prostaglandin D2
SerpinE1: Serine protease inhibitor clade E member 1
SM: Systemic mastocytosis
TGF-β1: Transforming growth factor-β1
TME: Tumor microenvironment
tPA: Tissue plasminogen activator
TSG101: Tumor susceptibility gene 101 protein
VCAM-1: Vascular cell adhesion molecule-1
YY1: Yin Yang 1
α-SMA: α-smooth muscle actin
